# P010: Bloodstream infections by drug-resistant organisms in a secondary hospital

**DOI:** 10.1186/2047-2994-2-S1-P10

**Published:** 2013-06-20

**Authors:** T Gimenez-Julvez, M Rodriguez-Aguirregabiria, C Campelo, E Palencia-Herrejon, MJ Moreno Sanchez, S de Juan-García

**Affiliations:** 1 Hospital Universitario Infanta Leonor, Spain; 2 BR-Salud, Madrid, Spain

## Introduction

Bloodstream infections (BSI) are important causes of morbidity and mortality. Most of all, when are caused by drug-resistant organisms (DR).

## Objectives

To investigate the epidemiology, etiology, systemic response and treatment of DR-BSI.

## Methods

A retrospective study was conducted about all BSI diagnosed in a secondary hospital during one year. The pattern resistant pathogen study was *EPINE-EPPS* project. Comparisons between groups were performed by means of the *X*^2^ test for categorical variables or analysis of variances (ANOVA) for continuous variables.

## Results

We included 60 patients [median and interquartile range (IQR) age, 73.5 years (60.5-79.5), 57.1% males, median (IQR) Charlson comorbidity index, 3 (2-4), median (IQR) acute physiology and chronic health evaluation (APACHE) II score, 11 (8-15)] with 63 DR-BSI of which 71.5% were nosocomial and healthcare-associated BSI.

Unknown and intravascular catheter-related DR-BSI accounted for 49.2% of cases. Among secundary infections, the source was 37.5% urinary track, 31.2% intra-abdominal and 15.6% respiratory track infections.

Overall DR-BSI, DR-Gram-positive cocci were 55.6%. The most common isolated pathogens were staphylococcus coagulase-negative and *S. aureus*. Among DR-Gram-negative bacilli, 12.2% of enterobacteracea family produced extended-spectrum B-lactamasas. We found 5 DR-BSI caused by *Acitetobacter* carbapenem resistant and 3 DR-BSI by *P. aeruginosa* carbapenem resistant.

Median time to diagnosis for DR-Nosocomial BSI was 14 days (IQR), 7-35 days after hospital admission. For Gram-negative was 11 days (7.5-31.5) and for Gram-positive 19 days (7-29).

Only 31.7% of DR-BSI received appropriate initial empirical antimicrobial therapy versus 73.5% of non DR-BSI (p<0.001). More than one third (36.5%) of the episodes occur with significant systemic response (severe sepsis or septic shock). The crude mortality rate was 25.4 % (p<0.001). If the patient developed severe sepsis or septic shock crude mortality rose to 52.2%.

## Conclusion

Information about local epidemiology is important to develop prevention and control strategies in drug–resistant microorganism and to improve the management of BSI.

## Disclosure of interest

None declared

